# Unified comparison of machine learning paradigms for blood transfusion prediction in pediatric congenital heart surgery

**DOI:** 10.1016/j.isci.2026.116181

**Published:** 2026-05-30

**Authors:** Ming-Wei Yin, Jing Li, Jian Huang, Zhu Zhu, Bao-Hai Chen, Zhuo Shi, Qian Jiang, Xue-Jun Chen, Gang Yu

**Affiliations:** 1 Department of Blood Transfusion, Children’s Hospital, Zhejiang University School of Medicine, National Clinical Research Center for Children and Adolescents’ Health and Diseases, Hangzhou, China; 2 Department of Data and Information, Children’s Hospital, Zhejiang University School of Medicine, National Clinical Research Center for Children and Adolescents’ Health and Diseases, Hangzhou, China; 3 Sino-Finland Joint AI Laboratory for Child Health of Zhejiang Province, Hangzhou, China; 4 Department of Cardiac Surgery, Children’s Hospital, Zhejiang University School of Medicine, National Clinical Research Center for Children and Adolescents’ Health and Diseases, Hangzhou, China

**Keywords:** Health sciences, Medicine, Medical specialty, Pediatrics

## Abstract

Blood transfusion prediction studies in pediatric cardiac surgery have employed direct regression, two-stage, and multi-class classification paradigms, but the fundamentally different outputs of these paradigms have prevented direct head-to-head comparison. We developed a dual-mean absolute error (MAE) composite metric that converts all predictions to continuous transfusion volume estimates, enabling unified comparison across paradigms. Using data from 3,342 pediatric patients undergoing congenital heart surgery, we evaluated six imputation methods, three class-balancing strategies, and fifteen machine learning algorithms. Iterative regression imputation achieved the highest performance (AUC = 0.864), and synthetic minority oversampling technique (SMOTE) combined with undersampling improved F1 by 37% (RBC) and 19.5% (plasma). The two-stage approach yielded the best composite scores for RBC (0.791; Neural Network + Extra Trees) and plasma (0.755; AdaBoost + KNN), whereas multi-class LightGBM was optimal for platelets (0.652; AUC = 0.960). SHAP analysis identified cardiopulmonary bypass as the dominant predictor for RBC/plasma and Aristotle score for platelets. The framework supports evidence-based, blood-product-specific model selection.

## Highlights

• Dual-MAE composite metric enables comparison across transfusion prediction paradigms
• Iterative imputation + SMOTE/undersampling handle missing data and class imbalance
• Two-stage best for RBC and plasma; multi-class LightGBM best for platelets
• Generalizable six-step framework for surgical transfusion volume prediction

## Graphical abstract

**Figure:**
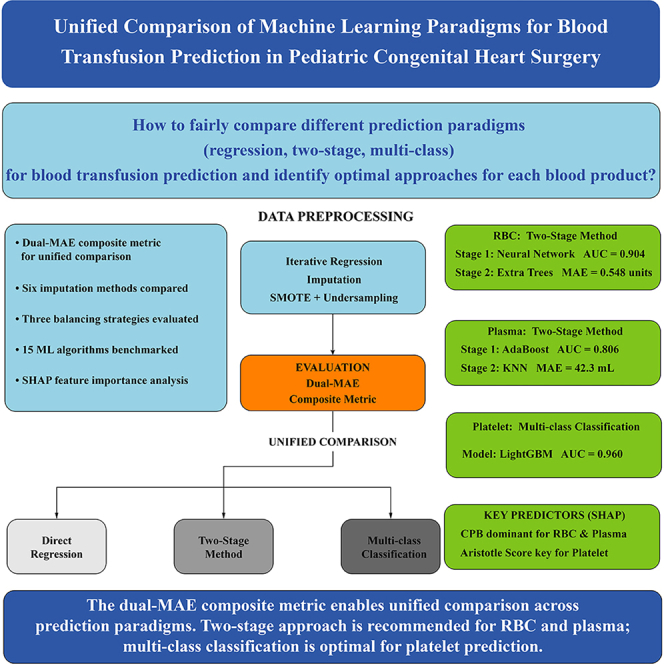

## Introduction

Blood transfusion is a critical component of perioperative management in pediatric congenital heart surgery, with transfusion rates ranging from 65% to 90% depending on surgical complexity and patient characteristics.^1^ The accurate prediction of blood transfusion requirements before surgery is essential for preoperative blood ordering decisions, enabling individualized blood bank resource allocation, reducing wastage, and improving patient outcomes. As artificial intelligence (AI) and machine learning (ML) technologies become increasingly widespread in medicine, many researchers have employed AI and ML to predict surgical blood transfusions.^2^^,^^3^^,^^4^ However, predicting transfusion needs in this population presents unique challenges due to the heterogeneity of congenital heart defects, varying surgical complexity, and the physiological differences between pediatric and adult patients.

Traditional approaches to blood ordering in cardiac surgery have relied on empirical guidelines and clinical experience, often resulting in over-ordering and subsequent wastage of blood products. Recent advances in ML have demonstrated promising results in predicting transfusion requirements across various surgical specialties.^5^ A comprehensive scoping review by Duranteau et al. systematically evaluated 40 studies using ML for surgical transfusion prediction, identifying hemoglobin, age, and platelet count as the most commonly used predictive variables, while highlighting logistic regression as the most frequently employed algorithm.^6^ Studies in adult cardiac surgery have shown promising results: Liu et al. compared 13 ML algorithms for predicting red blood cell (RBC) transfusion in aortic valve replacement surgery, achieving an AUC of 0.872 with logistic regression,^7^ while Wang et al. developed a multi-center prediction model for blood transfusion in mitral valve surgery across eight tertiary hospitals in China, with LightGBM achieving the best performance (AUC = 0.935).^8^ Furthermore, in our previous study, the Risk Adjustment for Congenital Heart Surgery (RACHS-1) score has been validated as a significant predictor of transfusion requirements, with higher scores associated with increased blood product utilization.^9^

Despite these advances, a critical gap exists in the current literature: the vast majority of ML studies on blood transfusion prediction have focused on adult populations, with pediatric cardiac surgery remaining a less extensively studied domain. Duranteau’s scoping review specifically noted the paucity of studies in pediatric populations, particularly in the context of congenital heart surgery where the heterogeneity of defects and unique physiological characteristics pose additional predictive challenges.^6^ Moreover, several methodological challenges remain inadequately addressed in the existing literature. First, missing data are prevalent in clinical datasets, yet the impact of different imputation strategies on model performance requires further evaluation.^8^^,^^10^ Second, class imbalance is particularly severe in blood transfusion prediction, especially for platelets transfusion where the transfusion rate may be less than 5%, leading to biased models with poor sensitivity. Third, and most critically, existing studies have employed diverse prediction paradigms, making direct comparison difficult. Some studies formulate transfusion prediction as a binary classification problem (transfusion vs. no transfusion), while others employ regression models to predict transfusion volume directly,^11^ and still others use multi-class classification to categorize transfusion amounts into discrete levels.^5^ Because these approaches produce fundamentally different outputs (probabilities, continuous values, and categorical labels), traditional evaluation metrics cannot compare them directly, leaving clinicians without evidence-based guidance for selecting the optimal prediction paradigm.

Our previous work on blood transfusion characteristics in congenital heart surgery identified age, weight, RACHS-1, and preoperative hemoglobin as independent predictors of transfusion.^9^ Building upon these findings, the present study aims to develop a comprehensive ML framework that systematically addresses these challenges. We further introduce a dual-MAE composite metric that enables fair comparison across fundamentally different prediction paradigms (direct regression, two-stage, and multi-class classification), providing evidence-based guidance for selecting the optimal approach for each blood product. Additionally, this study systematically compares six imputation methods for handling high missing rates, evaluates three balancing strategies for addressing severe class imbalance, comprehensively evaluates fifteen ML algorithms, and develops separate prediction models for three blood products (RBC, plasma, and platelets) to provide clinically actionable recommendations.

## Results

### High missing rates and severe class imbalance characterize the dataset

A total of 3,342 pediatric patients undergoing congenital heart surgery were included in this study. The baseline characteristics are summarized in Table 1. The median age was 25.7 months (interquartile range [IQR]: 7.8–56.7 months), and the median weight was 14.0 kg (IQR: 10.2–19.5 kg). Male patients accounted for 47.4% (*n* = 1,583) of the cohort. Regarding preoperative laboratory values, the median hemoglobin was 121.0 g/L (IQR: 113.0–128.0 g/L), RBC count was 4.4 × 10^12^/L (IQR: 4.1–4.7 × 10^12^/L), hematocrit was 36.1% (IQR: 33.8–38.1%), and platelet count was 314.0 × 10^9^/L (IQR: 263.0–373.8 × 10^9^/L). The median Aristotle score, reflecting surgical complexity, was 6.0 (IQR: 3.0–6.0). Cardiopulmonary bypass (CPB) was used in 66.3% (*n* = 2,216) of procedures.

**Table 1:** Baseline characteristics of the study population (*n* = 3,342)

Category	Variable	Statistics	Missing *n* (%)
**Demographics and clinical characteristics**	age (months)	25.7 (7.8–56.7)	0 (0.0%)
	weight (kg)	14.0 (10.2–19.5)	0 (0.0%)
	hemoglobin (g/L)	121.0 (113.0–128.0)	0 (0.0%)
	RBC count (×10^12^/L)	4.4 (4.1–4.7)	0 (0.0%)
	hematocrit (%)	36.1 (33.8–38.1)	0 (0.0%)
	platelet count (×10^9^/L)	314.0 (263.0–373.8)	0 (0.0%)
	fibrinogen (mg/dL)	1.9 (1.6–2.2)	325 (9.7%)
	albumin (g/L)	42.8 (40.6–44.8)	692 (20.7%)
	total protein (g/L)	65.4 (60.8–69.3)	692 (20.7%)
	direct bilirubin (μmol/L)	1.6 (1.1–2.4)	692 (20.7%)
	indirect bilirubin (μmol/L)	5.6 (4.0–8.6)	692 (20.7%)
	PT (seconds)	11.6 (11.0–12.2)	2,520 (75.4%)
	APTT (seconds)	30.0 (28.2–32.8)	2,520 (75.4%)
	Aristotle score	6.0 (3.0–6.0)	0 (0.0%)
**Categorical variables**	sex (male)	1,583 (47.4%)	0 (0.0%)
	CPB use	2,216 (66.3%)	0 (0.0%)
**Outcome variables (intraoperative transfusion)**	intraoperative RBC transfusion (units)	–	0 (0.0%)
	patients transfused, *n* (%)	593 (17.7%)	–
	patients not transfused, *n* (%)	2,749 (82.3%)	–
	amount if transfused, median (IQR)	1.0 (1.0–1.0)	–
	intraoperative plasma transfusion (mL)	–	0 (0.0%)
	patients transfused, *n* (%)	1,045 (31.3%)	–
	patients not transfused, *n* (%)	2,297 (68.7%)	–
	amount if transfused, median (IQR)	140.0 (120.0–210.0)	–
	intraoperative platelet transfusion (units)	–	0 (0.0%)
	patients transfused, *n* (%)	68 (2.0%)	–
	patients not transfused, *n* (%)	3,274 (98.0%)	–
	amount if transfused, median (IQR)	5.0 (5.0–5.0)	–
**Class imbalance (no transfusion: transfusion)**	RBC	4.64: 1	–
	plasma	2.20: 1	–
	platelet	48.15: 1	–

Continuous variables are presented as median (interquartile range, IQR). Categorical variables are presented as n (%). APTT, activated partial thromboplastin time; CPB, cardiopulmonary bypass; IQR, interquartile range; PT, prothrombin time; RBC, red blood cell. See also Table S1.

**Figure 1:**
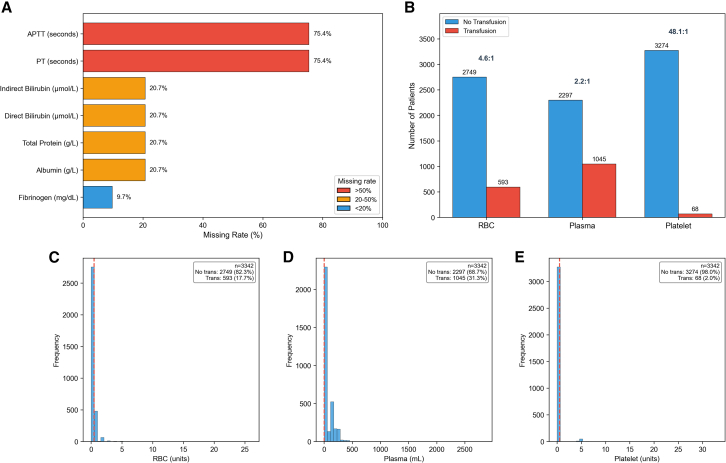
Characteristics of raw data: missing values and class imbalance (A) Missing rates of clinical variables. Variables are color-coded by missing severity: red (>50%), orange (20%–50%), and blue (<20%). APTT and PT showed the highest missing rates (75.4%), followed by bilirubin, total protein, and albumin (20.7%), with fibrinogen having the lowest missing rate (9.7%). (B) Class distribution and imbalance ratios for three blood products. The imbalance ratios were 4.6:1 for RBC, 2.2:1 for plasma, and 48.1:1 for platelets, indicating severe class imbalance particularly for platelets transfusion. (C–E) Distribution of transfusion volumes for RBC (C), plasma (D), and platelets (E). The red dashed lines indicate the threshold separating non-transfusion from transfusion cases. RBC transfusion occurred in 593 patients (17.7%), plasma in 1,045 patients (31.3%), and platelets in only 68 patients (2.0%). All three distributions showed pronounced right-skewness with zero-inflation, reflecting the predominance of non-transfusion cases. APTT, activated partial thromboplastin time; PT, prothrombin time; RBC, red blood cell.

The characteristics of the raw data revealed two major challenges: missing values and class imbalance (Figure 1). Missing data analysis showed that coagulation parameters (prothrombin time [PT] and activated partial thromboplastin time [APTT]) had the highest missing rates (each 75.4%, *n* = 2,520), followed by biochemical markers including albumin, total protein, direct bilirubin, and indirect bilirubin (each 20.7%, *n* = 692), and fibrinogen (9.7%, *n* = 325) (Figure 1A). In contrast, demographic variables, hematological parameters, and outcome variables had no missing data.

For intraoperative transfusion outcomes, 593 patients (17.7%) received RBC transfusion with a median volume of 1.0 unit (IQR: 1.0–1.0 unit) among transfused patients. Plasma transfusion was administered to 1,045 patients (31.3%) with a median volume of 140.0 mL (IQR: 120.0–210.0 mL). Platelets transfusion was the least common, occurring in only 68 patients (2.0%) with a median volume of 5.0 units (IQR: 5.0–5.0 units). The class imbalance was particularly severe for platelets transfusion, with a no-transfusion to transfusion ratio of 48.15:1 (3,274 vs. 68 patients), compared to 4.64:1 for RBC (2,749 vs. 593) and 2.20:1 for plasma (2,297 vs. 1,045) (Figure 1B). The distribution of transfusion volumes for each blood product exhibited marked right-skewness, with the majority of patients receiving no transfusion (Figures 1C–1E).

### Iterative regression imputation outperforms other methods

**Figure 2:**
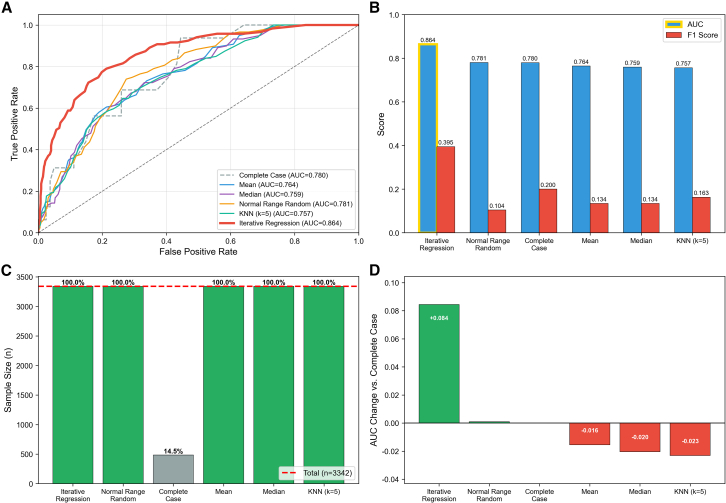
Comparison of six imputation methods for handling missing data (A) Receiver operating characteristic (ROC) curves for each imputation method. Iterative regression (red) achieved the highest AUC of 0.864, substantially outperforming other methods. The gray dashed diagonal line represents random classification. (B) Performance comparison showing AUC (blue) and F1 score (red) for each method. Iterative regression achieved both the highest AUC (0.864) and F1 score (0.395), while other methods showed comparable AUC values (0.757–0.781) but markedly lower F1 scores (0.104–0.200). (C) Sample size retention across imputation methods. Complete case analysis retained only 14.5% of the original cohort (*n* = 484), whereas all other imputation methods preserved the full dataset (*n* = 3,342, 100%), as indicated by the red dashed reference line. (D) AUC change relative to complete case analysis. Iterative regression demonstrated the largest improvement (+0.084), normal range random showed minimal change (+0.001), while mean, median, and KNN imputation resulted in slight performance decreases (−0.016 to −0.023). See also Table S2. AUC, area under the receiver operating characteristic curve; F1 score, the harmonic mean of precision and recall; ROC, receiver operating characteristic.

Six imputation methods were systematically compared for their impact on model performance in predicting RBC transfusion using Random Forest classification (Figure 2). Complete case analysis retained only 484 patients (14.5% of the total sample), resulting in substantial information loss. Among the imputation methods evaluated, iterative regression achieved the highest AUC (0.864) and F1 score (0.395), representing a marked improvement of 0.084 in AUC compared to complete case analysis (AUC = 0.780, F1 = 0.200). Normal range random imputation showed marginal improvement over complete case analysis (AUC = 0.781, F1 = 0.104), while mean imputation (AUC = 0.764, F1 = 0.134), median imputation (AUC = 0.759, F1 = 0.134), and KNN imputation (AUC = 0.757, F1 = 0.163) performed worse than complete case analysis in terms of AUC. Notably, all imputation methods preserved the full sample size (*n* = 3,342, 100%), whereas complete case analysis suffered from significant sample reduction. Based on these results, iterative regression imputation was selected for all subsequent analyses.

### SMOTE combined with undersampling optimizes F1 scores

**Figure 3:**
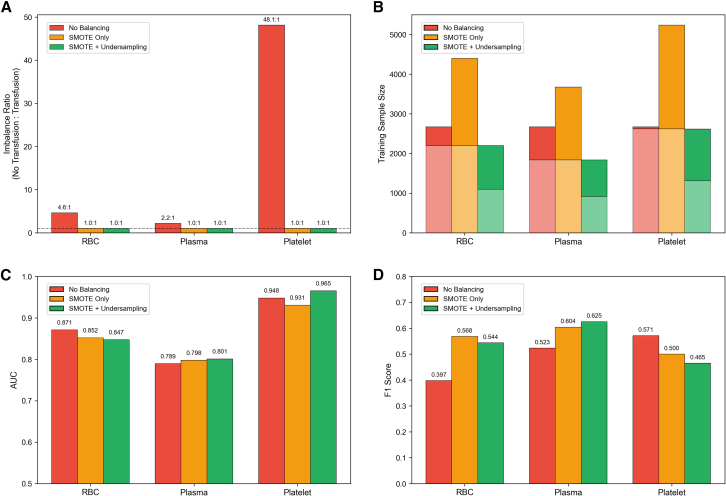
Comparison of three class balancing strategies for handling imbalanced data (A) Class imbalance ratios before and after balancing. The original imbalance ratios were 4.6:1 for RBC, 2.2:1 for plasma, and 48.1:1 for platelets. Both SMOTE only and SMOTE combined with undersampling achieved balanced distributions (1.0:1) across all blood products. The gray dashed line indicates perfect balance. (B) Training sample size after applying each balancing strategy. SMOTE only increased sample sizes substantially through synthetic oversampling, while SMOTE combined with undersampling maintained moderate sample sizes by simultaneously reducing majority class samples. (C) AUC performance across balancing strategies. No balancing achieved the highest AUC for RBC (0.871) and platelets (0.948), while SMOTE combined with undersampling performed best for plasma (0.801). AUC differences between strategies were relatively modest. (D) F1 score performance across balancing strategies. SMOTE combined with undersampling achieved the highest F1 scores for RBC (0.544) and plasma (0.625), representing improvements of 37% and 19.5% over no balancing, respectively. For platelets, no balancing yielded the highest F1 score (0.571), likely due to the extreme original imbalance ratio. AUC, area under the receiver operating characteristic curve; F1 score, the harmonic mean of precision and recall; RBC, red blood cell; SMOTE, synthetic minority oversampling technique.

The effect of class balancing strategies on model performance varied across blood products (Figure 3). All balancing strategies successfully achieved a 1:1 class ratio from the original imbalanced ratios of 4.6:1 for RBC, 2.2:1 for plasma, and 48.1:1 for platelets. For RBC prediction, no balancing resulted in high AUC (0.871) but poor F1 score (0.397). Synthetic minority oversampling technique (SMOTE) alone improved F1 to 0.568 while slightly reducing AUC to 0.852, and SMOTE combined with undersampling achieved F1 = 0.544 with AUC = 0.847, representing a 37% improvement in F1 over no balancing. For plasma prediction, SMOTE combined with undersampling achieved the best balance with AUC = 0.801 and F1 = 0.625, a 19.5% improvement in F1 over no balancing (AUC = 0.789, F1 = 0.523). For platelets prediction, the extremely severe imbalance presented unique challenges. No balancing achieved the highest AUC (0.948) with moderate F1 (0.571), while SMOTE alone reduced both metrics (AUC = 0.931, F1 = 0.500), and SMOTE combined with undersampling yielded the highest AUC (0.965) but the lowest F1 (0.465). Based on the overall performance across all three blood products, SMOTE combined with undersampling was selected as the balancing strategy for subsequent analyses.

### Two-stage method achieves optimal performance for RBC and plasma, multi-class for platelets prediction

**Figure 4:**
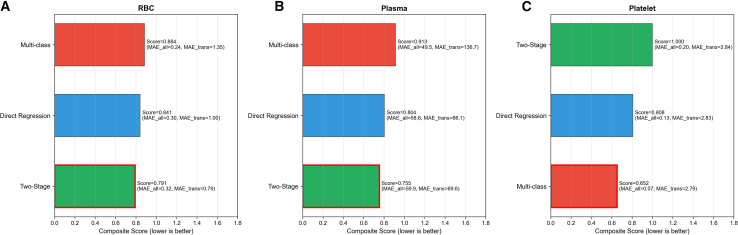
Comparison of three prediction approaches using a composite evaluation metric for blood transfusion prediction (A) Composite score integrating overall prediction accuracy (MAE_all, mean absolute error on all samples) and volume prediction accuracy for transfused patients (MAE_transfused, mean absolute error on transfused patients only) was calculated as: Composite Score = 0.5 × normalized_MAE_all +0.5 × normalized_MAE_transfused, where normalized MAE = MAE/max (MAE) within each blood product. Lower scores indicate better performance. (A) RBC prediction: two-stage achieved the lowest composite score (0.791), with MAE_all = 0.32 units and MAE_transfused = 0.79 units. (B) Plasma prediction: two-stage achieved the lowest composite score (0.755), with MAE_all = 59.9 mL and MAE_transfused = 69.6 mL. (C) Platelets prediction: multi-class achieved the lowest composite score (0.652), with MAE_all = 0.07 units and MAE_transfused = 2.79 units. Red borders indicate the best-performing method for each blood product. Methods are ranked from best (bottom) to worst (top) by composite score. Blue: direct regression; green: two-stage; red: multi-class. See also Table 2 and Figure S3. Sample size: test set *n* = 669; transfused subset for RBC *n* = 119, plasma *n* = 209, platelet *n* = 14. RBC, red blood cell.

Three prediction approaches were evaluated using a composite metric that integrated overall prediction accuracy (MAE on all samples) and volume prediction accuracy for transfused patients (MAE on transfused patients only) (Figure 4; Table 2). All approaches were converted to continuous transfusion volume predictions for fair comparison: direct regression output was truncated at zero, two-stage predicted zero for stage 1 negatives and regression values for positives, and multi-class predictions were converted to training set category median values.

**Table 2:** Benchmark comparison: traditional vs. dual-MAE framework-selected optimal approaches

Blood product	Approach	Best algorithm	MAE (all)	MAE (transfused)	Composite score	Optimal	Improvement vs. baseline	Stage 1 AUC	Stage 2 MAE/R^2^
RBC	direct regression	Random Forest	0.298 units	1.004 units	0.841	–	Baseline	0.865	—
	two-stage	NN + Extra Trees	0.318 units	0.785 units	0.791	✓	+5.9%	0.904	0.548 (R^2^ = 0.461)
	multi-class	Neural Network	0.244 units	1.349 units	0.884	–	−5.1%	0.904	—
Plasma	direct regression	Neural Network	58.6 mL	86.1 mL	0.804	–	Baseline	0.840	—
	two-stage	AdaBoost + KNN	59.9 mL	69.6 mL	0.755	✓	+6.1%	0.806	42.3 mL (R^2^ = 0.256)
	multi-class	AdaBoost	49.5 mL	136.7 mL	0.913	–	−13.6%	0.806	—
Platelet	direct regression	Ridge	0.126 units	2.832 units	0.808	–	Baseline	0.948	—
	two-stage	LightGBM + —	0.203 units	2.844 units	1.000	–	−23.8%	—	—
	multi-class	LightGBM	0.066 units	2.786 units	0.652	✓	+19.3%	0.960	—

Composite score = (Normalized MAE_all + Normalized MAE_transfused)/2, Lower is better; ✓ indicates the optimal approach selected by dual-MAE framework; two-stage = Classification (Stage 1) + Regression (Stage 2); for platelet, two-stage method shows “—” because the extremely low transfusion rate (2.0%) resulted in insufficient samples for stage 2 regression. AUC, area under the receiver operating characteristic curve; MAE, mean absolute error; R^2^, coefficient of determination; RBC, red blood cell; NN, Neural Network; two-stage, classification (stage 1) plus regression (stage 2).

For RBC prediction, two-stage achieved the lowest composite score (0.791), compared to direct regression (0.841) and multi-class (0.884), representing a 5.9% improvement over the traditional approach (Table 2; Figure 4A). Multi-class achieved the lowest MAE on all samples (0.24 units), while two-stage achieved the lowest MAE among transfused patients (0.79 units vs. 1.00 and 1.35 units for direct regression and multi-class, respectively). The optimal algorithm combination was Neural Network for stage 1 classification (AUC = 0.904) and Extra Trees for stage 2 regression (MAE = 0.548 units). For plasma prediction, two-stage achieved the lowest composite score (0.755), compared to direct regression (0.804) and multi-class (0.913), representing a 6.1% improvement (Table 2; Figure 4B). Multi-class achieved the lowest MAE on all samples (49.5 mL), while two-stage achieved substantially lower MAE among transfused patients (69.6 mL vs. 86.1 mL for direct regression and 136.7 mL for multi-class). The optimal combination was AdaBoost for classification (AUC = 0.806) and KNN for regression (MAE = 42.3 mL). For platelets prediction, multi-class achieved the lowest composite score (0.652), compared to direct regression (0.808) and two-stage (1.000), representing a 19.3% improvement (Table 2; Figure 4C). Multi-class achieved the lowest MAE on all samples (0.07 units) and comparable MAE among transfused patients (2.79 units vs. 2.83 units for direct regression and 2.84 units for two-stage). LightGBM was the optimal algorithm (AUC = 0.960). Table 2 summarizes the benchmark comparison across all prediction paradigms with specific algorithms used for each approach.

### Neural Network plus Extra Trees optimal for RBC, AdaBoost plus KNN for plasma, and LightGBM for platelet prediction

**Figure 5:**
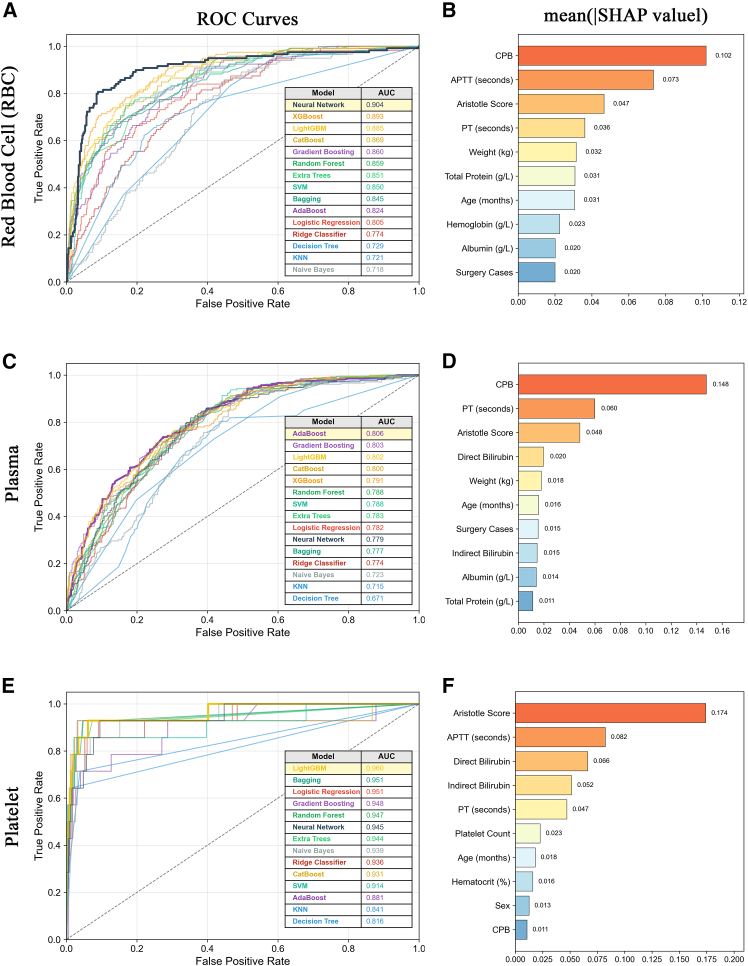
Machine learning model performance and SHAP feature importance analysis for blood transfusion prediction. The optimal prediction approach was used for each blood product based on Figure 4 results: two-stage method for RBC and plasma, multi-class classification for platelets (A, C, and E) ROC curves comparing 15 ML algorithms for RBC (A), plasma (C), and platelets (E) prediction. Inset tables display model performance ranked by AUC in descending order. For RBC, Neural Network achieved the highest AUC (0.904), followed by XGBoost (0.893) and LightGBM (0.885). For plasma, AdaBoost achieved the highest AUC (0.806), followed by Gradient Boosting (0.803) and LightGBM (0.802). For platelets, LightGBM achieved the highest AUC (0.960), followed by Bagging (0.951) and logistic regression (0.951). (B, D, and F) SHAP (SHapley Additive exPlanations) feature importance analysis showing top 10 predictive features for RBC (B), plasma (D), and platelets (F), derived from Random Forest classification. Mean absolute SHAP values represent the average contribution of each feature to model predictions. For RBC, CPB (0.102), and APTT (0.073) were the most important predictors. For plasma, CPB (0.148) and PT (0.060) were dominant. For platelets, Aristotle score (0.174) and APTT (0.082) showed substantially higher importance than other features, while CPB (0.011) contributed minimally. The 15 algorithms evaluated include: Logistic Regression, Ridge Classifier, Decision Tree, Random Forest, Extra Trees, Bagging, AdaBoost, Gradient Boosting, XGBoost, LightGBM, CatBoost, SVM, KNN, Naive Bayes, and Neural Network. See also Figures S1 and S2. Sample sizes: training *n* = 2,673; test *n* = 669 (transfused *n* = 119/209/14 for RBC/plasma/platelet, respectively). APTT, activated partial thromboplastin time; AUC, area under the receiver operating characteristic curve; CPB, cardiopulmonary bypass; ML, machine learning; PT, prothrombin time; RBC, red blood cell; SHAP, SHapley Additive exPlanations; SVM, support vector machine; KNN, *k*-nearest neighbors.

Model performance was evaluated on the held-out 20% test set. Using the optimal prediction approach for each blood product (two-stage for RBC and plasma, multi-class for platelets), 15 ML algorithms were evaluated (Figure 5). For RBC prediction using the two-stage method, Neural Network achieved the highest AUC (0.904), followed by XGBoost (0.893), LightGBM (0.885), CatBoost (0.869), and Gradient Boosting (0.860) (Figure 5A). Logistic regression (0.805) and Ridge Classifier (0.774) showed moderate performance, while Decision Tree (0.729), KNN (0.721), and Naive Bayes (0.718) showed lower performance. SHAP analysis identified CPB as the most important predictor (mean |SHAP value| = 0.102), followed by APTT (0.073), Aristotle score (0.047), PT (0.036), and weight (0.032) (Figure 5B). For plasma prediction using the two-stage method, AdaBoost achieved the highest AUC (0.806), followed by Gradient Boosting (0.803), LightGBM (0.802), and CatBoost (0.800) (Figure 5C). Most ensemble methods achieved AUC values between 0.77 and 0.81. SHAP analysis revealed CPB as the dominant predictor (mean |SHAP value| = 0.148), substantially outweighing other features including PT (0.060), Aristotle score (0.048), direct bilirubin (0.020), and Weight (0.018) (Figure 5D). For platelets prediction using multi-class classification, LightGBM achieved the highest AUC (0.960), followed by Bagging (0.951), logistic regression (0.951), Gradient Boosting (0.948), and Random Forest (0.947) (Figure 5E). Most algorithms achieved AUC > 0.90. SHAP analysis identified Aristotle score as the most important predictor (mean |SHAP value| = 0.174), followed by APTT (0.082), direct bilirubin (0.066), indirect bilirubin (0.052), and PT (0.047) (Figure 5F). Notably, CPB showed minimal importance for platelets prediction (0.011), in contrast to its dominant role in RBC and plasma prediction.

**Figure 6:**
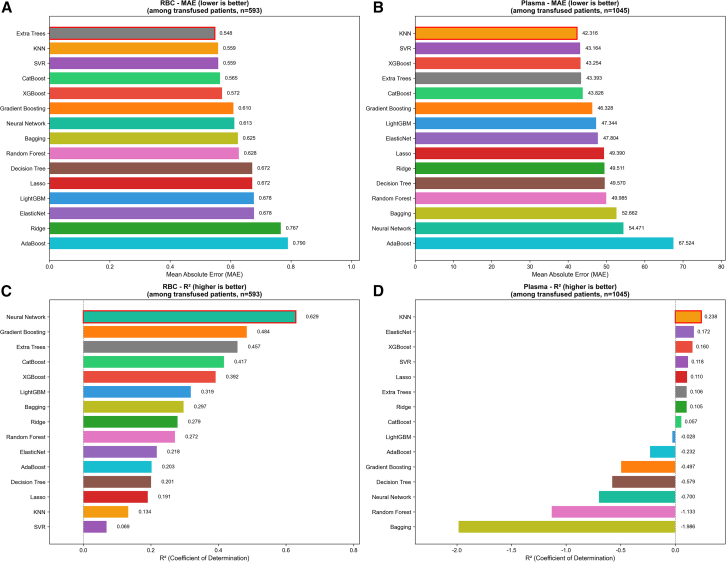
Regression performance comparison of 15 machine learning algorithms for transfusion volume prediction (two-stage method, stage 2). This analysis evaluates the second stage of the two-stage prediction approach, where regression models are trained exclusively on transfused patients to predict actual transfusion volume (A) MAE (mean absolute error) comparison for RBC (*n* = 593 transfused patients). Extra Trees achieved the lowest MAE (0.548 units), followed by KNN (0.559) and SVR (0.559). (B) MAE comparison for plasma (*n* = 1,045 transfused patients). KNN achieved the lowest MAE (42.3 mL), followed by SVR (43.2 mL) and XGBoost (43.3 mL). Algorithms are ranked from best (top) to worst (bottom). (C) R^2^ (coefficient of determination) comparison for RBC. Neural Network achieved the highest R^2^ (0.629), followed by Gradient Boosting (0.484) and Extra Trees (0.457). (D) R^2^ comparison for plasma. KNN achieved the highest R^2^ (0.238), followed by ElasticNet (0.172) and XGBoost (0.160). Several algorithms showed negative R^2^ values, including Bagging (−1.986), Random Forest (−1.133), and Neural Network (−0.700), indicating poor model fit. Red borders indicate the best-performing algorithm in each panel. The 15 regression algorithms evaluated include: Ridge, Lasso, ElasticNet, Decision Tree, Random Forest, Extra Trees, Bagging, AdaBoost, Gradient Boosting, XGBoost, LightGBM, CatBoost, SVR, KNN, and Neural Network. See also Figure S1. Sample sizes for stage-2 regression: RBC *n* = 593 transfused patients; plasma *n* = 1,045 transfused patients. MAE, mean absolute error; RBC, red blood cell; R^2^, coefficient of determination; SVR, support vector regression; KNN, *k*-nearest neighbors.

For the two-stage method, 15 regression algorithms were evaluated in stage 2 for transfusion volume prediction among transfused patients (Figure 6). For RBC (*n* = 593), Extra Trees achieved the lowest MAE (0.548 units), followed by KNN (0.559) and SVR (0.559) (Figure 6A). Neural Network achieved the highest R^2^ (0.629), followed by Gradient Boosting (0.484) and Extra Trees (0.457), indicating moderate to good explanatory power (Figure 6C). For plasma (*n* = 1,045), KNN achieved the lowest MAE (42.3 mL), followed by SVR (43.2 mL) and XGBoost (43.3 mL) (Figure 6B). R^2^ values were substantially lower than RBC prediction, with KNN achieving the highest R^2^ (0.238), followed by ElasticNet (0.172) and XGBoost (0.160). Several algorithms showed negative R^2^ values, including Bagging (−1.986), Random Forest (−1.133), Neural Network (−0.700), and Decision Tree (−0.579), indicating poor model fit for plasma volume prediction (Figure 6D).

## Discussion

This study presents a comprehensive ML framework for predicting blood transfusion requirements in pediatric congenital heart surgery. A key methodological contribution is a dual-MAE composite metric that enables fair comparison across fundamentally different prediction paradigms. While existing studies have employed diverse approaches including binary classification, direct regression, and multi-class classification, their fundamentally different outputs have limited direct comparison using conventional evaluation metrics. Our dual-MAE metric addresses this gap by converting all predictions to continuous volume estimates and calculating both overall accuracy and transfused-patient-specific accuracy. The systematic evaluation of 3,342 pediatric patients across six imputation methods, three class balancing strategies, three prediction approaches, and fifteen ML algorithms provides evidence-based guidance for developing clinical decision support tools.

The comparison of data preprocessing strategies yielded important methodological insights. Iterative regression imputation substantially outperformed simpler approaches, with an AUC improvement of 0.084 over complete case analysis (0.864 vs. 0.780), underscoring the critical importance of appropriate missing data handling in clinical datasets. Although using only complete cases (*n* = 484, 14.5%) avoids imputation assumptions, it results in substantial information loss and potential selection bias, as patients with coagulation test results may represent a non-representative subgroup. Complete case analysis using the same analytical framework yielded consistent paradigm recommendations for RBC and platelet, but with lower model performance across all blood products, further supporting the imputation approach (Table S2). For class imbalance, SMOTE combined with undersampling improved F1 scores by 37% for RBC and 19.5% for plasma while maintaining acceptable AUC values, consistent with previous research.^8^^,^^12^ Our benchmark comparison demonstrates that the optimal prediction approach is outcome-dependent (Table 2): two-stage achieved the best composite score for RBC (0.791, 5.9% improvement over direct regression) and plasma (0.755, 6.1% improvement), while multi-class was optimal for platelets (0.652, 19.3% improvement). The two-stage method excels when sufficient transfused samples (RBC *n* = 593; plasma *n* = 1,045) allow meaningful regression in stage 2, while multi-class is advantageous for highly imbalanced outcomes like platelets (2.0% transfusion rate). Notably, for platelets, multi-class functionally approximated binary classification due to the extreme concentration of transfusion volumes at 5 units (69.1%). These specific paradigm recommendations are inherently dataset-dependent; the primary contribution is the dual-MAE unified comparison framework itself, which researchers can apply to their own cohorts to derive institution-specific recommendations. Our two-stage rationale is consistent with recent methodological advances: Jalali et al. found gradient boosting performed well for both classification and regression in pediatric craniofacial surgery,^11^ and Wang et al. developed a similar hybrid approach for cardiothoracic surgery.^13^

Among the 15 ML algorithms evaluated, Neural Network achieved the highest accuracy for RBC prediction (AUC = 0.904), AdaBoost for plasma (AUC = 0.806), and LightGBM for platelets prediction (AUC = 0.960). For RBC prediction, these results are comparable to or exceed those reported in adult cardiac surgery studies: Cunha et al. achieved an AUC of 0.83 using gradient boosting in Brazilian cardiac surgery patients,^14^ while Benjamin et al. reported AUC values of 0.81 for massive transfusion prediction.^15^ For plasma prediction, our AdaBoost model (AUC = 0.806) demonstrated competitive performance; notably, Perduca et al. used 137 ML algorithms to predict post-CPB coagulation parameters including prothrombin ratio and fibrinogen in adult cardiac surgery, achieving accurate predictions for coagulation factors that guide plasma transfusion decisions.^16^ For platelets prediction, our LightGBM model achieved an exceptionally high AUC of 0.960, which substantially exceeds the performance reported by Engelke et al., whose deep learning model for platelets transfusion prediction in cardiothoracic surgery achieved an AUC-PR of only 0.42, attributed to the unpredictable nature of intraoperative bleeding events.^17^ Additionally, Guo et al. developed ML models to predict postoperative coagulation state in children with congenital heart disease, demonstrating the feasibility of ML approaches in pediatric cardiac surgery populations.^18^ The finding that different algorithms achieved optimal performance for different blood products suggests that a one-size-fits-all approach is inappropriate and provides practical guidance for clinicians developing prediction tools for specific blood products. SHAP analysis revealed that CPB was the dominant predictor for RBC (mean |SHAP value| = 0.102) and plasma (0.148) transfusion. Notably, CPB was not included in our previous study using traditional logistic regression,^9^ which may partly explain the improved predictive performance observed in the current study. The strong predictive value of CPB is consistent with its well-established role in hemodilution, activation of inflammatory cascades, and coagulation disturbances. Aristotle score, reflecting surgical complexity, was the most important predictor for platelets transfusion (0.174) and consistently among the top three predictors across all blood products, aligning with our previous findings^9^ and other studies demonstrating surgical complexity as a dominant factor in determining transfusion requirements.^19^ Coagulation parameters (APTT and PT) also emerged as important predictors across all blood products, a finding not observed in our previous logistic regression study,^9^ likely reflecting the ability of ML algorithms to capture non-linear relationships^20^^,^^21^ and consistent with findings in other surgical populations.^22^^,^^23^ The high missing rate for these parameters (75.4%) highlights the need for future studies to prioritize complete ascertainment of coagulation data.

**Figure 7:**
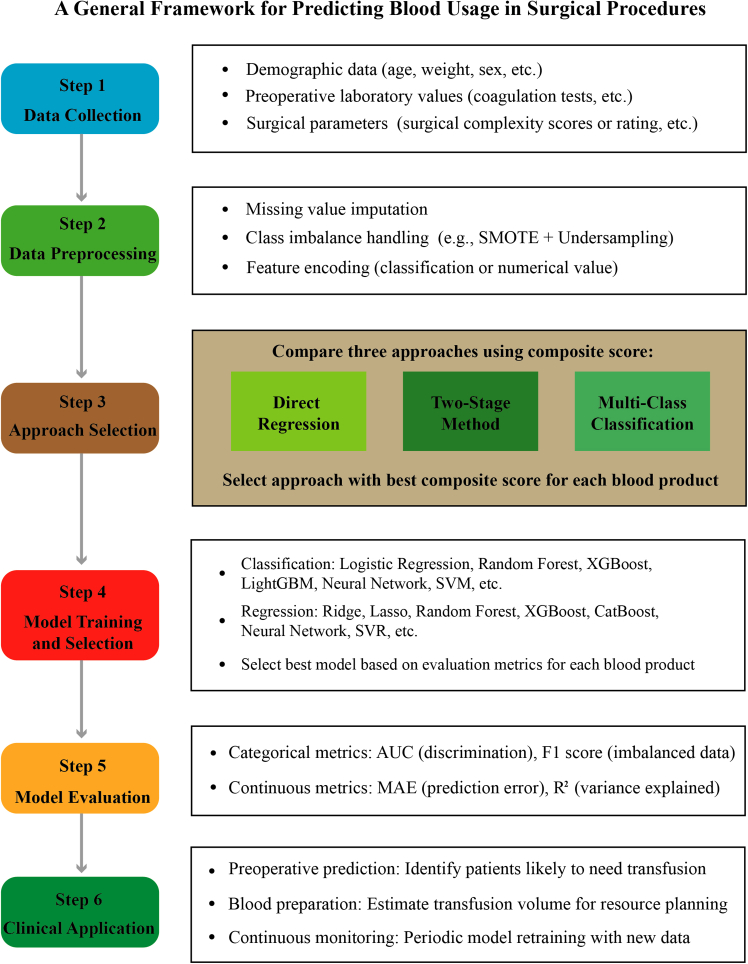
Modeling framework for blood transfusion prediction in surgical procedures (A) Generalizable workflow showing six steps: data collection, data preprocessing, prediction approach selection, model training and selection, model evaluation, and clinical application. AUC, area under the receiver operating characteristic curve; F1, F1 score; MAE, mean absolute error; R^2^, coefficient of determination; SMOTE, synthetic minority oversampling technique; SVM, support vector machine; SVR, support vector regression.

Based on our findings, we propose a generalizable six-step modeling framework for blood transfusion prediction in surgical procedures (Figure 7), encompassing data collection, preprocessing with systematic evaluation of imputation and balancing strategies, prediction approach comparison, model training and selection, comprehensive evaluation with feature importance analysis, and clinical application with continuous monitoring. In practice, this framework could be integrated into the hospital information system (HIS) to automatically extract the required preoperative variables from electronic medical records, generating individualized transfusion volume predictions for RBC, plasma, and platelets separately. These predictions can guide the blood bank in preparing patient-specific blood products, potentially reducing unnecessary crossmatching and wastage while ensuring adequate supply for patients predicted to require transfusion. Decision curve analysis confirmed positive net clinical benefit across broad threshold ranges for all three recommended models (Figure S3). This framework directly addresses the methodological gaps identified in recent reviews,^6^ which emphasized the need for standardized reporting of data preprocessing and model validation procedures. The modular design allows adaptation to different institutional contexts while maintaining methodological rigor, facilitating external validation and multi-center collaboration essential for clinical translation.

In summary, this study’s primary contribution is the development of a dual-MAE composite metric that enables fair comparison across fundamentally different prediction paradigms (direct regression, two-stage, and multi-class classification) for blood transfusion prediction. The benchmark comparison demonstrates that the two-stage approach achieves 5.9% (RBC) and 6.1% (plasma) improvement over direct regression, and 10.5% and 17.3% improvement over multi-class classification, respectively; while for platelets, multi-class classification achieves 19.3% improvement over direct regression and 34.8% improvement over the two-stage approach. Based on this unified evaluation framework in pediatric congenital heart surgery, we provide clear clinical recommendations: the two-stage approach is optimal for RBC (Neural Network + Extra Trees) and plasma (AdaBoost + KNN) prediction, while multi-class LightGBM is recommended for platelets prediction. For real-world clinical data challenges, iterative regression imputation and SMOTE combined with undersampling are recommended. Future research should focus on external validation and incorporation of real-time intraoperative data.

### Limitations of the study

Several limitations should be acknowledged. First, this is a single-center retrospective study. We employed a stratified random split (80/20) rather than temporal splitting to avoid confounders from evolving surgical techniques over the study period; the comparability of training and test sets was confirmed in Table S1, and training set performance is provided in Figure S1. However, external validation using independent multi-center cohorts remains essential for confirming generalizability. Second, the reliance on preoperative static variables to predict a dynamic intraoperative process is an inherent limitation, as actual transfusion decisions are influenced by real-time factors such as surgical blood loss and point-of-care testing results.^24^^,^^25^ Future research should explore the integration of real-time intraoperative data to develop dynamic prediction models. Third, probability calibration analysis showed good to excellent Brier scores (RBC: 0.103; plasma: 0.209; platelet: 0.016; Figure S2), though in clinical deployment, the operating threshold should be adjusted to prioritize sensitivity given the greater harm of under-preparation. Future work should also incorporate formal model uncertainty quantification and post hoc probability calibration techniques.

## Resource availability

### Lead contact

Requests for further information and resources should be directed to and will be fulfilled by the lead contact, Gang Yu (yugbme@zju.edu.cn).

### Materials availability

This study did not generate new unique reagents.

### Data and code availability

• The de-identified clinical data reported in this study have been deposited at Mendeley Data and are publicly available at https://doi.org/10.17632/776566vnmm.1.
• All original code is available as Data S1 in the supplemental information.
• Any additional information required to reanalyze the data reported in this paper is available from the lead contact upon request.

## Acknowledgments

This work was supported by the 10.13039/501100012166National Key R&D Program of China (grant number 2023YFC2706400).

## Author contributions

Conceptualization, M.-W.Y., X.-J.C., and G.Y.; methodology, M.-W.Y., J.L., and J.H.; software, M.-W.Y., J.L., J.H. and Z.Z.; formal analysis, M.-W.Y., J.L., and Z.Z.; investigation, M.-W.Y. and Z.S.; data curation, B.-H.C. and Q.J.; writing – original draft, M.-W.Y. and J.L.; writing – review and editing, X.-J.C. and G.Y.; visualization, M.-W.Y., J.L., and J.H.; supervision, X.-J.C. and G.Y.; funding acquisition, G.Y.

## Declaration of interests

The authors declare no competing interests.

## Declaration of generative AI and AI-assisted technologies in the writing process

During the preparation of this work, the author(s) used DeepSeek in order to assist with study design and methodology development, and ChatGPT (OpenAI) in order to assist with manuscript language editing and proofreading. After using these tools, the author(s) reviewed and edited the content as needed and take(s) full responsibility for the content of the publication.

## STAR★Methods

### Key resources table

**Table:** 

REAGENT or RESOURCE	SOURCE	IDENTIFIER
**Deposited data**		

Clinical data	This paper	Mendeley Data: https://doi.org/10.17632/776566vnmm.1

**Software and algorithms**		

Python 3.10	Python Software Foundation	https://www.python.org/; RRID:SCR_008394
scikit-learn	Pedregosa et al.^26^	https://scikit-learn.org/; RRID:SCR_002577
XGBoost	Chen and Guestrin^27^	https://xgboost.readthedocs.io/; RRID:SCR_025885
LightGBM	Ke et al.^28^	https://lightgbm.readthedocs.io/
CatBoost	Prokhorenkova et al.^29^	https://catboost.ai/
imbalanced-learn	Lemaitre et al.^30^	https://imbalanced-learn.org/
SHAP	Lundberg and Lee^31^	https://shap.readthedocs.io/; RRID:SCR_025891
matplotlib	Hunter^32^	https://matplotlib.org/; RRID:SCR_008624

**Other**		

Original code for this paper	This paper	Data S1

### Experimental model and study participant details

This retrospective cohort study included 3,342 consecutive pediatric patients (a Chinese pediatric population; median age 25.7 months [IQR 7.8–56.7]; 47.4% male, 52.6% female) who underwent congenital heart surgery at Children’s Hospital, Zhejiang University School of Medicine from 2019 to 2023. The study was approved by the Ethics Committee of Children’s Hospital, Zhejiang University School of Medicine, National Clinical Research Center for Children and Adolescents’ Health and Diseases (approval No. 2023-IRB-0287-P-01). This study was conducted in accordance with the Declaration of Helsinki and informed consent was waived owing to the retrospective design. Patients were included if they had documented preoperative laboratory values and intraoperative transfusion records. Patients undergoing emergency surgery were excluded. Baseline characteristics are summarized in Table 1. Sex was included as a predictor in every model and evaluated through SHAP feature-importance analysis; the influence of sex on transfusion outcomes was small relative to surgical complexity and CPB use, and is reported in Figures 5B, 5D, and 5F.

### Method details

#### Data collection

Preoperative variables collected included demographic data (age in months, weight in kg, sex), laboratory values (hemoglobin, RBC count, hematocrit, platelet count, fibrinogen, albumin, total protein, direct and indirect bilirubin, prothrombin time [PT], and activated partial thromboplastin time [APTT]), surgical complexity score (Aristotle Score), and surgical parameters (cardiopulmonary bypass [CPB] use, procedure type, number of surgical procedures). The primary outcomes were intraoperative transfusion volumes for three blood products: RBC (in units), plasma (in mL), and platelets (in units).

#### Missing data analysis and imputation

Missing data patterns were analyzed for all predictor variables. Patients with complete data comprised only 484 cases (14.5% of total sample). Variables with missing rates included fibrinogen (9.7%), albumin, total protein, direct bilirubin, and indirect bilirubin (each 20.7%), and PT and APTT (each 75.4%). Six imputation methods were compared: (1) complete case analysis, which included only patients with complete data; (2) mean imputation, where missing values were replaced with variable means; (3) median imputation, where missing values were replaced with variable medians; (4) normal range random imputation, where missing values were randomly sampled from clinically normal ranges; (5) KNN imputation (k = 5), where missing values were imputed based on similar patients; and (6) iterative regression imputation, where missing values were estimated through iterative multivariate regression. The imputation methods (2)–(6) assume that missing data are Missing at Random (MAR), which is reasonable in this context as the variables with high missing rates were missing primarily because these tests were not routinely ordered for all patients, rather than being related to transfusion outcomes. Each imputation method was evaluated based on downstream model performance using Random Forest classification for RBC transfusion prediction, with AUC and F1 score as primary metrics.

#### Class imbalance handling

The dataset exhibited significant class imbalance across all three blood products. For RBC, the no-transfusion to transfusion ratio was 4.6:1 (2,749 vs. 593 patients); for plasma, the ratio was 2.2:1 (2,297 vs. 1,045 patients); and for platelets, the imbalance was most severe at 48.1:1 (3,274 vs. 68 patients). To address this challenge, three balancing strategies were compared: (1) no balancing, where the original imbalanced data were used directly for model training; (2) SMOTE only, where the Synthetic Minority Over-sampling Technique was applied to generate synthetic samples and achieve a 1:1 ratio between classes; and (3) SMOTE combined with undersampling, where SMOTE was first applied with a sampling strategy of 0.5 to increase minority class samples, followed by random undersampling of the majority class with a sampling strategy of 1.0 to achieve balanced classes.

#### Unified evaluation framework for prediction approach selection

To enable fair comparison across three fundamentally different prediction approaches (Direct Regression, Two-Stage, and Multi-class classification), all predictions were converted to continuous transfusion volume estimates. For Direct Regression, predicted values were truncated at zero to prevent negative predictions. For the Two-Stage approach, patients predicted as non-transfusion in Stage 1 were assigned zero, while those predicted as requiring transfusion received the Stage 2 regression output. For multi-class classification, each predicted category was converted to the median transfusion volume of that category calculated from the training set. Multi-class thresholds were determined based on the distribution characteristics of transfusion volumes in the study cohort. For RBC, thresholds were set at 0.5, 2, and 4 units, reflecting the concentration of transfusion volumes around 1 unit (80.9% of transfused patients) and the clinical distinction between standard (≤2 units) and large-volume (>4 units) transfusion. For Plasma, thresholds at 50, 150, and 300 mL corresponded to natural groupings in the volume distribution and approximate packaging specifications (typically around 100 mL and 200 mL per bag). For Platelet, due to the extremely low transfusion rate (2.0%) and high concentration of volumes at 5 units (69.1% of transfused patients), the multi-class approach effectively identified transfusion occurrence, with volume estimated by the training set class median.

Two complementary Mean Absolute Error (MAE) metrics were calculated for each approach:

$${MAE}_{all}=\frac{1}{n}\sum_{i=1}^{n}|y_{i}-{ŷ}_{i}|$$

$${MAE}_{transfused}=\frac{1}{n_{t}}\sum_{i\in T}|y_{i}-{ŷ}_{i}|$$

where *n* is the total number of test samples, *n*_*t*_ is the number of transfused patients in the test set, *T* is the set of transfused patients, *y*_*i*_ is the actual transfusion volume, and *ŷ*_*i*_ is the predicted transfusion volume.

*MAE*_*all*_ reflects overall prediction accuracy including the ability to correctly identify zero-transfusion cases, while *MAE*_*transfused*_ specifically evaluates volume prediction accuracy among patients who received transfusion, which is more clinically relevant for blood preparation.

To integrate these two metrics into a single composite score, *MAE* values were first normalized within each blood product by dividing by the maximum *MAE*:

$$normalize\_MAE=\frac{MAE}{\max\left(MAE\right)}$$

The composite score was then calculated as the weighted average:

$$Composite\;Score=w1\times normalized\_{MAE}_{all}+w2\times normalized\_{MAE}_{transfused}$$

where *w*1 = *w*2 = 0.5, giving equal weight to overall accuracy and transfused patient accuracy. Lower composite scores indicate better overall performance. The prediction approach with the lowest composite score was selected as optimal for each blood product.

This composite metric serves as a methodological comparison framework to support comparison across prediction paradigms: previous studies have employed direct regression, two-stage, and multi-class classification approaches for transfusion prediction, but their fundamentally different outputs made direct comparison impossible using traditional metrics alone. By converting all predictions to continuous volume estimates, the Dual-MAE composite enables unified evaluation across paradigms. *MAE*_*all*_ captures the model’s utility for blood bank resource planning (including correctly assigning zero volume to non-transfusion cases), while *MAE*_*transfused*_ reflects the model’s utility for ensuring adequate blood preparation for patients who require transfusion. The composite score is used solely for paradigm selection; once the optimal approach is identified for each blood product, the clinical application follows the selected method directly (e.g., Two-Stage for RBC and Plasma, Multi-class for Platelet).

#### Machine learning models

All 16 preoperative variables were included as model inputs, and three separate prediction models were developed independently for each blood product (RBC, plasma, and platelets). All available variables were included rather than performing prior variable selection, as tree-based and ensemble ML algorithms inherently handle irrelevant features; post-hoc SHAP analysis was used to identify the most important predictors. Fifteen ML algorithms were evaluated for both classification and regression tasks, covering a comprehensive range of model architectures. Linear models included Logistic Regression with L2 regularization for classification, and Ridge Regression, Lasso Regression, and ElasticNet for regression. Tree-based approaches employed Decision Tree with a maximum depth of 10. Bagging-based ensemble methods included Random Forest, Extra Trees, and Bagging, each with 100 estimators. Boosting-based ensemble methods comprised AdaBoost, Gradient Boosting, XGBoost, LightGBM, and CatBoost, with 100 estimators or iterations. Additional models included Support Vector Machine/Regression with radial basis function kernel, KNN with k = 5 and distance weighting, Gaussian Naive Bayes (classification only), and Neural Network implemented as a multi-layer perceptron with two hidden layers (100 and 50 neurons, ReLU activation, Adam optimizer). Prior to training, all continuous features were standardized using *Z* score normalization. For the Two-Stage approach, regression algorithms were applied to predict transfusion volume among patients identified as requiring transfusion in the first stage.

#### Model evaluation

Models were evaluated using a stratified train-test split with 80% training and 20% testing data, stratified by binary transfusion status to ensure proportional representation of transfused and non-transfused cases. The preprocessing pipeline followed the order: missing data imputation on the full dataset, train-test split, *Z* score standardization (fitted on training data and applied to test data), and class balancing (applied to training data only). Classification performance was assessed using area under the receiver operating characteristic curve (AUC) and F1 score (the harmonic mean of precision and recall). For regression tasks in the Two-Stage approach, Mean Absolute Error (MAE) and coefficient of determination (R^2^) were calculated to evaluate volume prediction accuracy among transfused patients. AUC was used as the primary metric for model comparison due to its threshold-independent nature and clinical interpretability.

#### Feature importance analysis

The importance of each predictor variable (feature) was assessed using SHAP (SHapley Additive exPlanations) values derived from Random Forest models. Mean absolute SHAP values, which quantify the average contribution of each predictor variable to model predictions, were calculated to identify the top 10 most important features for each blood product (RBC, plasma, and platelets).

### Quantification and statistical analysis

Continuous variables were presented as mean ± standard deviation or median (interquartile range [IQR]) as appropriate based on data distribution. Categorical variables were presented as counts and percentages. Comparisons of baseline characteristics between the training and test sets were performed using the Mann-Whitney U test for continuous variables and the chi-squared test for categorical variables; statistical significance was set at *p* < 0.05 (two-sided). Data were randomly split into 80% training and 20% testing sets, stratified by binary transfusion status, with a fixed random seed (random_state = 42) for reproducibility. Classification performance was assessed using AUC and F1 score. Regression performance was assessed using MAE and R^2^. The Dual-MAE composite score was used for unified comparison across prediction paradigms, as described in Method details. All analyses were performed using Python 3.10 with scikit-learn (v1.2)^26^ for model development and evaluation; XGBoost (v1.7),^27^ LightGBM (v3.3),^28^ and CatBoost (v1.1)^29^ for gradient boosting algorithms; imbalanced-learn (v0.10)^30^ for SMOTE and undersampling; SHAP (v0.42)^31^ for feature importance analysis, and matplotlib (v3.6)^32^ for visualization.

## Supplemental information

Document S1. Figures S1–S3 and Tables S1 and S2

Data S1. Original analysis code used for data preprocessing, model training, evaluation, and visualization
