# Supplementary material for: Unified comparison of machine learning paradigms for blood transfusion prediction in pediatric congenital heart surgery
Source: iScience. 2026 May 30;29(6):116181. doi: 10.1016/j.isci.2026.116181 (PMC13241783; doi:10.1016/j.isci.2026.116181)
Supplement: Document S1. Figures S1–S3 and Tables S1 and S2 [file mmc1.pdf]

iScience, Volume *29*

Supplemental information

Unified comparison of machine learning paradigms for blood transfusion prediction in pediatric congenital heart surgery

Ming-Wei Yin, Jing Li, Jian Huang, Zhu Zhu, Bao-Hai Chen, Zhuo Shi, Qian Jiang, Xue-Jun Chen, and Gang Yu

Supplementary Figure S1. Training set performance for machine learning models in blood transfusion prediction

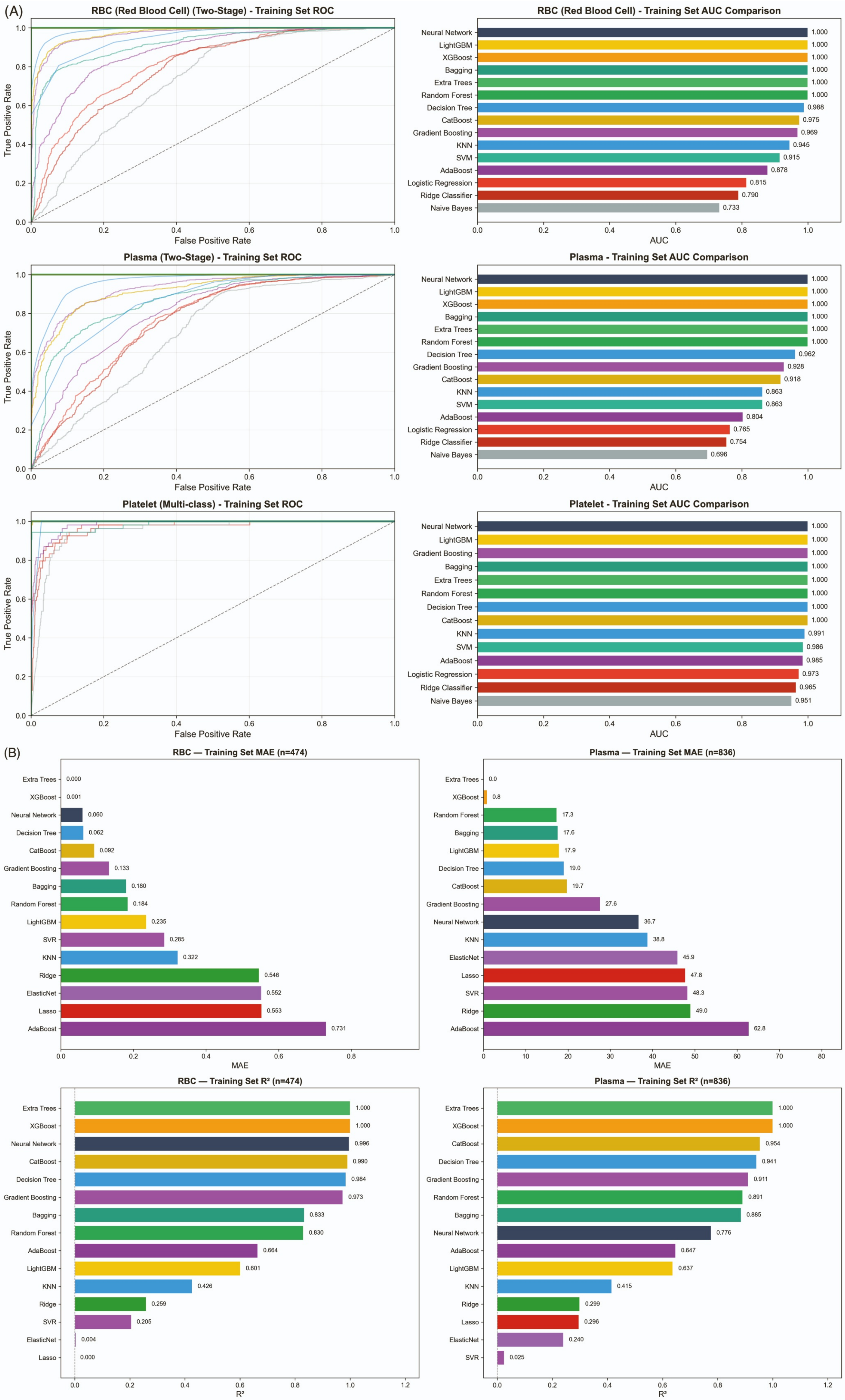

Training set performance was evaluated on the balanced data after SMOTE combined with undersampling to assess model fitting capacity. (A) Classification model performance on the balanced training set, showing ROC curves and AUC values for 15 machine learning algorithms for RBC (Two-Stage method), Plasma (Two-Stage method), and Platelet (Multi-class method) prediction. Multiple tree-based ensemble methods achieved perfect AUC of 1.000 on the balanced training data. Linear models and boosting methods showed lower training AUC values, suggesting less overfitting and potentially better generalization. (B) Regression model performance on the training set for the Two-Stage method Stage 2, showing MAE and R^2 comparisons for RBC (n=474) and Plasma (n=836) volume prediction among transfused patients. Note: Test set performance reported in the main text was evaluated on the original imbalanced data to reflect real-world generalization capability. Sample sizes: training set n=2,673 (balanced to n=4,398 after SMOTE + undersampling); Stage-2 regression on transfused training patients only (RBC n=474; plasma n=836).

Supplementary Figure S2. Probability calibration analysis of recommended classification models for transfusion prediction

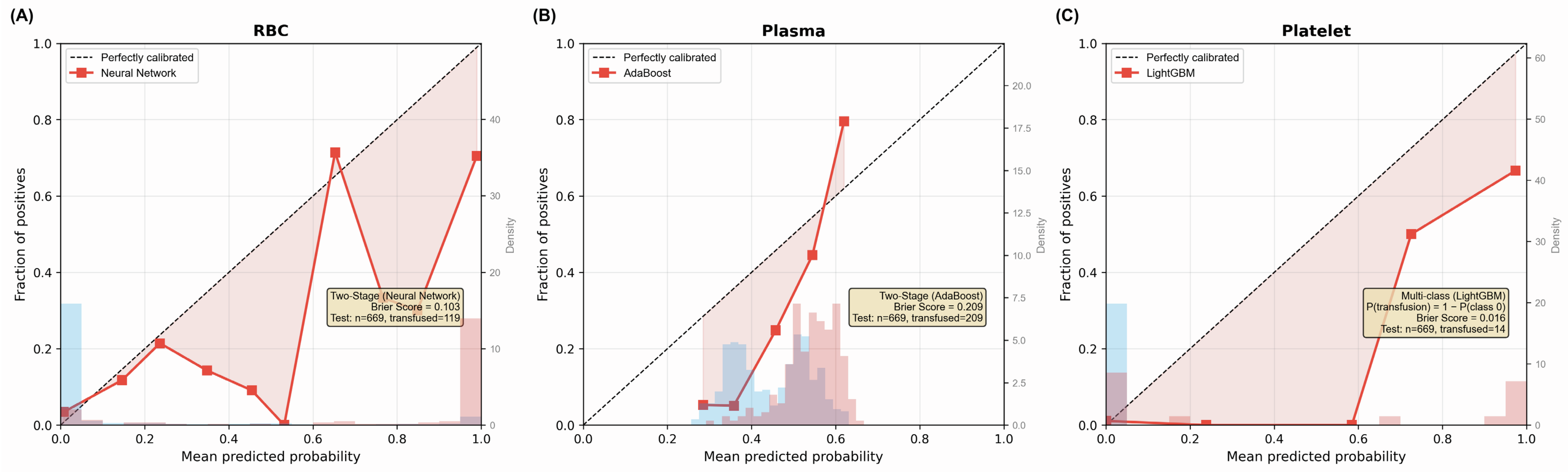

Calibration curves (reliability diagrams) and Brier scores assessing whether predicted transfusion probabilities reliably reflect actual transfusion likelihood. For RBC and Plasma (Two-Stage method), Stage 1 binary classification probabilities were evaluated directly. For Platelet (Multi-class method), multi-class probabilities were converted to binary: P(transfusion) = 1 − P(class 0). Background histograms show the distribution of predicted probabilities for non-transfusion (blue) and transfused (red) patients. (A) RBC: Neural Network achieved good calibration (Brier Score = 0.103, n=669, transfused=119). (B) Plasma: AdaBoost showed moderate calibration (Brier Score = 0.209, n=669, transfused=209), with predicted probabilities concentrated in a narrower range characteristic of boosting algorithms. (C) Platelet: LightGBM achieved excellent overall calibration (Brier Score = 0.016, n=669, transfused=14), though the extreme class imbalance limits the reliability of the calibration curve. The dashed diagonal line represents perfect calibration. Red shading indicates deviation from perfect calibration. Brier scores range from 0 (perfect) to 1 (worst). This analysis complements the volume prediction accuracy (MAE) reported in Table 2, together providing a comprehensive assessment of both the "whether to transfuse" and "how much to prepare" decisions.

Supplementary Figure S3. Decision curve analysis (DCA) of recommended models for transfusion prediction

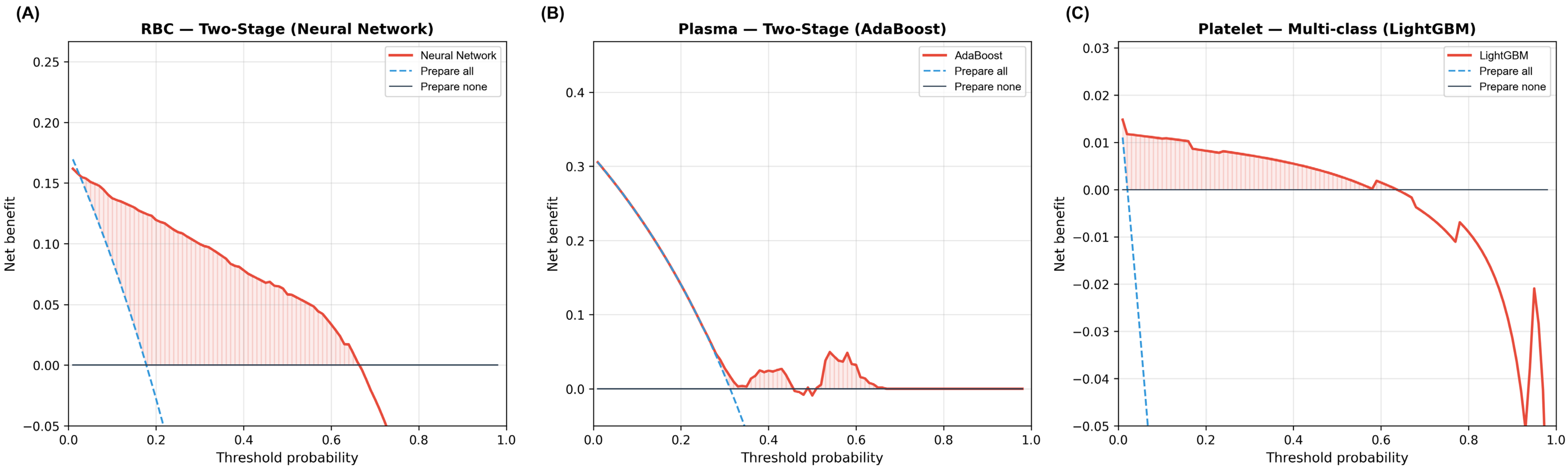

DCA evaluates the net clinical benefit of using the prediction model compared to two default strategies: "prepare blood for all patients" (blue dashed line) and "prepare for none" (black line). The red line represents the recommended model. The x-axis represents the threshold probability at which a clinician would decide to prepare blood; the y-axis represents the net benefit. (A) RBC: Neural Network (Two-Stage) provided net benefit over default strategies across threshold probabilities of 0.03–0.66. (B) Plasma: AdaBoost (Two-Stage) provided net benefit across thresholds of 0.07–0.66. (C) Platelet: LightGBM (Multi-class) provided net benefit across thresholds of 0.01–0.63. All three models demonstrated positive net clinical benefit across a broad range of decision thresholds, supporting their practical utility for preoperative blood ordering decisions. Sample size: test set n=669 (transfused: RBC n=119, plasma n=209, platelet n=14). Net benefit was computed without statistical inference; threshold ranges over which each recommended model exceeded both default strategies are reported.

Supplementary Table S1. Comparison of baseline characteristics between the training set (n=2,673) and test set (n=669)

Variable	Training Set (n=2,673)	Test Set (n=669)	P value
Demographics and Clinical Characteristics			
Age (months)	25.3 (7.6-56.6)	26.6 (9.0-58.8)	0.246
Weight (kg)	14.0 (10.0-19.5)	14.0 (10.8-19.5)	0.316
Hemoglobin (g/L)	121.0 (112.0-128.0)	121.0 (114.0-129.0)	0.134
RBC Count ($\times 10^{12}$/L)	4.4 (4.1-4.7)	4.5 (4.2-4.7)	0.031
Hematocrit (%)	36.1 (33.7-38.0)	36.3 (34.2-38.4)	0.102
Platelet Count ($\times 10^{9}$/L)	313.0 (262.0-373.0)	317.0 (267.0-375.0)	0.338
Fibrinogen (mg/dL)	1.9 (1.6-2.2)	2.0 (1.7-2.3)	0.001
Albumin (g/L)	42.9 (41.1-44.4)	43.0 (41.2-44.7)	0.546
Total Protein (g/L)	65.6 (61.5-69.0)	66.0 (62.1-69.8)	0.080
Direct Bilirubin (μmol/L)	1.7 (1.2-2.5)	1.6 (1.1-2.4)	0.187
Indirect Bilirubin (μmol/L)	5.8 (3.9-9.6)	5.7 (3.9-9.0)	0.437
PT (seconds)	11.8 (11.5-12.2)	11.8 (11.5-12.2)	0.641
APTT (seconds)	31.6 (29.7-34.0)	31.6 (29.6-33.9)	0.402
Aristotle Score	6.0 (3.0-6.0)	4.5 (3.0-6.0)	0.013
Categorical Variables			
Sex (Male)	1243 (46.5%)	340 (50.8%)	0.050
CPB Use	1779 (66.6%)	437 (65.3%)	0.577
Outcome Variables (Intraoperative Transfusion)			
RBC Transfusion			
Patients transfused, n (%)	474 (17.7%)	119 (17.8%)	1.000
Amount if transfused, median (IQR)	1.0 (1.0-1.0)	1.0 (1.0-2.0)	
Plasma Transfusion			
Patients transfused, n (%)	845 (31.6%)	200 (29.9%)	0.418
Amount if transfused, median (IQR)	140.0 (120.0-210.0)	140.0 (120.0-220.0)	
Platelet Transfusion			
Patients transfused, n (%)	57 (2.1%)	11 (1.6%)	0.518
Amount if transfused, median (IQR)	5.0 (5.0-5.0)	5.0 (5.0-5.5)	

Continuous variables are presented as median (interquartile range, IQR). Categorical variables are presented as n (%). P values were calculated using the Mann-Whitney U test for continuous variables and the chi-squared test for categorical variables. APTT, activated partial thromboplastin time; CPB, cardiopulmonary bypass; IQR, interquartile range; PT, prothrombin time; RBC, red blood cell.

Supplementary Table S2. Complete case analysis: prediction paradigm comparison and 15-algorithm classification performance (n=484)

Part A. Three-paradigm comparison with Dual-MAE Composite Scores

Blood Product	Method	MAE (All)	MAE (Transfused)	Composite Score
RBC	Direct Regression	0.360	1.389	0.919
RBC	Two-Stage	0.307	1.015	0.734
RBC	Multi-class	0.294	1.656	0.909
Plasma	Direct Regression	69.830	155.879	0.805
Plasma	Two-Stage	78.327	137.371	0.817
Plasma	Multi-class	56.082	216.667	0.858
Platelet	Direct Regression	0.346	2.787	0.811
Platelet	Two-Stage	0.440	2.137	0.821
Platelet	Multi-class	0.206	3.333	0.734

Regression algorithm comparison was not performed due to insufficient transfused patients in the test set (RBC: approximately 16; Platelet: approximately 3).

Part B. 15 classification algorithm comparison (AUC and F1)

Algorithm	RBC AUC	RBC F1	Plasma AUC	Plasma F1	Platelet AUC	Platelet F1
Logistic Regression	0.747	0.450	0.744	0.507	0.837	0.167
Ridge Classifier	0.755	0.426	0.719	0.500	0.879	0.333
Decision Tree	0.733	0.471	0.607	0.435	0.796	0.333
Random Forest	0.843	0.526	0.690	0.438	0.902	0.364
Extra Trees	0.783	0.375	0.664	0.455	0.901	0.444
Bagging	0.816	0.512	0.655	0.448	0.784	0.364
AdaBoost	0.748	0.526	0.682	0.452	0.922	0.250
Gradient Boosting	0.797	0.486	0.635	0.419	0.943	0.364
XGBoost	0.802	0.564	0.660	0.492	0.872	0.364
LightGBM	0.814	0.424	0.638	0.413	0.897	0.200
CatBoost	0.762	0.465	0.718	0.514	0.922	0.286
SVM	0.752	0.478	0.679	0.451	0.770	0.267
KNN	0.739	0.392	0.606	0.411	0.766	0.222
Naive Bayes	0.756	0.276	0.654	0.409	0.887	0.133
Neural Network	0.597	0.216	0.604	0.424	0.865	0.286

Composite Score = (Normalized MAE_all + Normalized MAE_transfused)/2; lower is better. The same analytical framework (SMOTE + Undersampling, stratified 80/20 split, random_state=42) was applied as in the main analysis. AUC, area under the receiver operating characteristic curve; F1, F1 score; MAE, mean absolute error; RBC, red blood cell; SVM, support vector machine; KNN, k-nearest neighbors; NN, neural network.
